# Food and waterborne protozoan parasites: The African perspective

**DOI:** 10.1016/j.fawpar.2020.e00088

**Published:** 2020-09-09

**Authors:** Joyce Siwila, Florence Mwaba, Nozyechi Chidumayo, Chishimba Mubanga

**Affiliations:** a Department of Clinical Studies, School of Veterinary Medicine, University of Zambia, P.O. Box 32379, Lusaka, Zambia; b Department of Pathology and Microbiology, School of Medicine, University of Zambia, P.O. Box 50110, Lusaka, Zambia; c Ministry of Agriculture, P.O. Box 31658, Lusaka, Zambia

**Keywords:** *Cryptosporidium*, *Giardia*, *Cyclospora*, *Entamoeba*, Food- and waterborne parasites, Africa

## Abstract

Parasitic food-borne diseases, particularly those caused by the protozoan parasites *Cryptosporidium*, *Giardia*, *Cyclospora cayetanensis* and *Entamoeba* are increasingly becoming common and have received considerable attention in the last two decades. The ability of the transmission stages of the parasites to survive in the environment for prolonged periods, globalization of the food industry and changes in eating habits have contributed to the numbers of human infections. This systematic scoping review highlights these important water- and foodborne parasites in the African context, detailing the burden in African water sources, wastewater/effluents and fresh produce. A scoping review search targeting African countries was conducted in Medline, Web of science and African journals online as well as back referencing from included studies covering the period 1990 to January 2020. Out of 1134 studies, 68 were included in the review. The articles covered 17 out of 54 African countries. There were 39/68 studies reporting on water sources while the rest reported on fresh produce. *Cryptosporidium* prevalence ranged from 6 to 100% in surface water, 4 to 100% in tap water and up to 100% in wastewater and sludge. In fresh produce, *Cryptosporidium* was reported from five countries with prevalence of 0.8–75%. *Giardia* was reported in 47 out of 68 articles; prevalence ranged from 2.4% in surface water; 1% to over 70% in tap water; 28–100% in wastewater and 2% - 99% in fresh produce. Prevalence of *Cyclospora cayetanensis* was lower. Prevalence of *Entamoeba* was 78% in surface water; 100% in wastewater and up to 99% in fresh produce. This study finds that Africa is no exception to the risk presented by the subject parasites from water and/or food sources. Routine screening for these parasites particularly at household level and provision of adequate and safe drinking water would help to control the parasites.

## Highlights

• African rural communities faced with clean water challenges.
• African water bodies and sources contaminated with *Cryptosporidium*, *Giardia*, *Cyclospora*, and *Entamoeba* and other pathogens
• Fresh produce are carriers of protozoan parasites *Cryptosporidium*, *Giardia*, *Cyclospora*, *Entamoeba*.

## Graphical abstract

**Figure:**
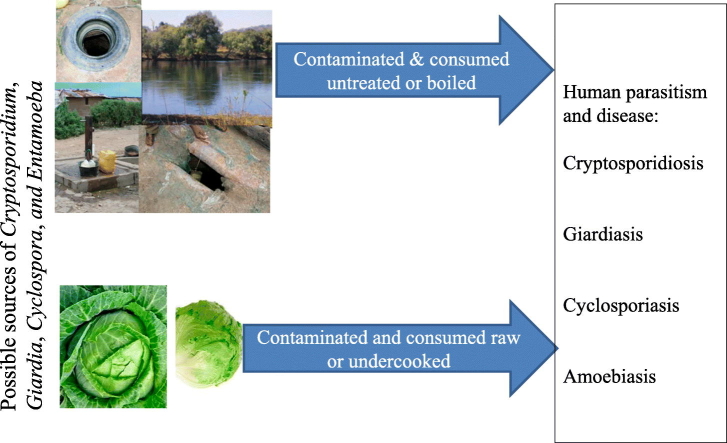

## 1 Introduction

Parasitic protozoa are ubiquitous in nature and have a (Abd El-Salam, 2012) worldwide distribution. They are responsible for epidemic and endemic human suffering in both the developed and developing countries (Cotruva et al., 2004). Some parasites are zoonotic in nature, therefore, occur in animals (Robertson and Gjerde, 2001); their occurrence on foods and water should be considered a public health concern. Food and waterborne parasites are generally under-recognised, but this scenario has changed over the years due to a number of food and water borne disease outbreaks attributed to parasites (Marshall et al., 1997). In the past, the risk of human infection with parasites was considered to be limited to distinct geographic regions due to food eating practices, parasites' adaptations to specific definitive hosts and intermediate hosts and environmental conditions (Orlandi et al., 2002; El Said Said, 2012). This is slowly changing due to the globalization of the food supply and availability of refrigerated food transport which has made it easier to transport various foods across borders (Dorny et al., 2009). Despite the inability of the parasitic protozoa to multiply in foods, they may survive in or on moist foods for months in cool (such as refrigerator) or damp environments, making it possible to convey infective stages of the parasite from one point to another (Dawson, 2005). This is especially true for *Cryptosporidium* oocysts and *Giardia* cysts which were shown to survive well when kept moist and refrigerated, at 4 °C (Utaaker et al., 2017).

Most of the sub-Saharan African population still lives without access to clean water and sanitary facilities (Sente et al., 2016) and in many African households, especially the rural dwellers, untreated water is used for various purposes including drinking, cooking, washing of fruits and other fresh produce, bathing, and swimming which exposes them to not only protozoan parasites but other pathogens as well (Yongsi, 2010; WHO, 2014). The possible contamination of drinking water with protozoan pathogens therefore poses a serious threat to millions of people in the developing world. Reports from periodic diarrhoeal disease outbreaks in the developed world have commonly been attributed to protozoan parasites such as *Cryptosporidium* sp. and *Giardia duodenalis* and to a lesser extent, *Cyclospora cayetanensis* and *Entamoeba histolytica* (Lane and Lloyd, 2002; Karanis et al., 2007), with *Cryptosporidium* species being responsible for majority of them. Most of the protozoan parasites use the faecal-oral route of transmission and infection can be direct (human to human, animal to human) or indirectly through contaminated drinking and recreational water, food and food products contaminated with infectious (oo) cysts or inhalation (Fayer et al., 2000; Ethelberg et al., 2009; Sponseller et al., 2014). Supplementary Table 1 (suppl1) summarises the parasites' transmission routes, brief life cycle, clinical signs and treatment. Very little is known about these important parasites with regard to food and water contamination in Africa, particularly sub-Saharan Africa. The faecal-oral route of transmission implies that humans can be infected through sewage contamination of drinking water sources by animal or human faeces (Lanata, 2003).

Other than limited access to clean water and sanitary facilities, other factors equally significantly contribute to the risk of transmission of food and waterborne parasites, especially among the highly susceptible individuals such as the aged, young children, malnourished and HIV infected (Dorny et al., 2009; Lund and O'Brien, 2012). Increase in the eating of raw and undercooked foods (Dorny et al., 2009) and eating food from street food vendors or open markets' caterers who do not always respect food safety probably play a significant role in the transmission of these parasites in the African setting (Kudah et al., 2018).

*Cryptosporidium* is an apicomplexan, intracellular, zoonotic parasite that causes cryptosporidiosis, a diarrhoeal disease of humans and animals (Fayer, 2010). It is frequently reported in waterborne outbreaks (Karanis et al., 2007). According to the World Health Organization, *Cryptosporidium* is the most common diarrhoea causing protozoan parasite worldwide (WHO/UNICEF, 2009). Severity and duration of symptoms are influenced by age, immune and nutritional status of the host, infective dose and genetics of the parasite. On the other hand, *Giardia duodenalis* is a protozoan flagellate that infects mammals including humans, and accounts for more than 250 million symptomatic human infections annually, with the less developed countries being the most afflicted (Einarsson et al., 2016). The parasite is a species complex, comprising eight genetic assemblages A-H (Caccio and Ryan, 2008). Assemblage A and B infects humans and other animals while C & D infects canids, E – hooved animals, F – cats, G rats and H – marine mammals (Minetti et al., 2016; Caccio et al., 2017). However, human infections with assemblages E and F have recently been reported, highlighting the zoonotic potential of the parasite (Abdel-Moein and Saeed, 2016; Fantinatti et al., 2016; Gelanew et al., 2007; Zahedi et al., 2017). Assemblages A and B are further subdivided into sub-assemblages AI, AII, AIII, AIV, BI, BII, BIII & BIV (Monis et al., 2003). *Giardia duodenalis* is commonly isolated in humans, with prevalence ranging from <10% (in developed countries) to up to around 30% or higher in countries with resource poor communities (Feng and Xiao, 2011; Einarsson et al., 2016). It is considered the most common protozoan parasite infecting the human intestines, causing an estimated 280 million cases each year (Lane and Lloyd, 2002). *Giardia* has been associated with several water- and foodborne outbreaks worldwide (Karanis et al., 2007; Baldursson and Karanis, 2011).

*Cyclospora cayetanensis*, another apicomplexan parasite, is an important waterborne parasite in industrialized countries (Herwaldt, 2000; Insulander et al., 2010) but it is likely that its effect on human health is underestimated. The parasite causes the disease cyclosporiasis. Host's age and immune status and endemicity in a particular region appear to influence severity of infection (Giangaspero and Gasser, 2019). Outbreaks of cyclosporiasis have increasingly been observed since the 1990s, particularly in North America and Asia. In 1996, more than 1000 cases, associated with eating raspberries, occurred in the United States of America and Canada (Herwaldt et al., 1997). Other outbreaks were associated with consumption of lettuce (CDC, 1997), or fresh basil (Hoge et al., 1995). During the period 2013–2014, over 900 people in the US were infected with *C. cayetanensis* linked to imported cilantro and salad mixes (CDC, 2014; Harvey, 2013). Because of these reports, the parasite has been considered of public health concern primarily in developed countries. *Entamoeba*, another protozoan parasite belongs to the phylum Sarcomastigophora, subphylum Sarcodina and has many species such as *Entamoeba histolytica*, *E. dispar*, *E. coli*, *E. hartmanni*, *E. gingivalis*, *E. polecki*, *E. moshkovskii*, *Dientamoeba fragilis*, *Endolimax nana* and *Iodamoeba bütschlii*. Among these, some are pathogenic to humans while others are commensals (Carrero et al., 2020). In this review, only *E. histolytica*/*dispar* is discussed. Even though clinical diagnosis of amoebiasis usually depends on the visualization of parasites by light microscopy of a wet smear or stained specimens, trophozoites and cysts of the pathogenic *Entamoeba histolytica* and non-pathogenic *E. dispar* are morphologically indistinguishable. Species identification requires use of molecular methods. Young age, pregnancy, malignancy, malnutrition, alcoholism, and prolonged corticosteroid use are some of the risk factors; and high-risk groups include travellers, immigrants, migrant workers, refugees, immunocompromised individuals, institutionalized individuals and, possibly, children in day-care centres (Salit et al., 2009; Carrero et al., 2020).

Despite the public health importance of these parasites, which has not extensively been highlighted in Africa, little is known about the occurrence and prevalence in food and water in Africa, the common modes through which infections occur.

### 1.1 The scoping review question

The subject protozoan parasites can infect humans from a variety of sources which can be broadly categorized as environment, animals and human sources. Studies looking at associations between risk factors and disease often sample humans to measure the rate of disease in relation to the selected risk factors. These studies do not detail the rate of occurrence of parasites in environmental and animal sources. This information is obtained when the individual potential sources of infection are sampled. Despite the many negative health effects of the four parasites (*Cryptosporidium*, *Giardia*, *Cyclospora cayetanensi*s and *Entamoeba*), very little has been done to highlight the prevalence of the parasites in water and fresh produce in Africa. Additionally, limited studies have been conducted to determine the occurrence of the parasite in fresh produce which have been reported to be common vehicles for several parasites (Robertson and Gjerde, 2001). The aim of this review is therefore to provide an overview of evidence of *Cryptosporidium*, *Giardia*, *Cyclospora cayetanensis* and *Entamoeba* contamination on fruits and vegetables that are consumed raw and various water sources (and effluents) in Africa and highlight their potential for water and foodborne disease.

## 2 Methods

The study was conducted according to the methodology by the Joana Brigs Institute (JBI) (Peters et al., 2015) and the reporting guidelines of the Preferred Reporting System for Systematic Reviews and Meta-Analysis for scoping review Protocols (PRISMA ScR) (Tricco et al., 2018).

### 2.1 Inclusion criteria

Primary studies meeting the following criteria were included in this ScR; (1) all primary studies on the occurrence of the four parasites *Cryptosporidium*, *Giardia*, *Cyclospora cayetanensis* and *Entamoeba* in various water bodies/sources and fresh produce particularly fruits and vegetables with samples from Africa (any of the 54 countries), (2). study design - all peer reviewed observation studies (3) studies published from1990 up to January 2020 with no language restriction.

### 2.2 Exclusion criteria

This review excluded (1) editorials, systematic reviews or reviews covering the subject parasites with no primary data, (2) studies on the subject parasites but with a different population such as humans, animals, other environmental samples besides water, (3) samples outside Africa or with different outcomes and studies outside the targeted time frame.

### 2.3 Search strategy

**Fig. 1:**
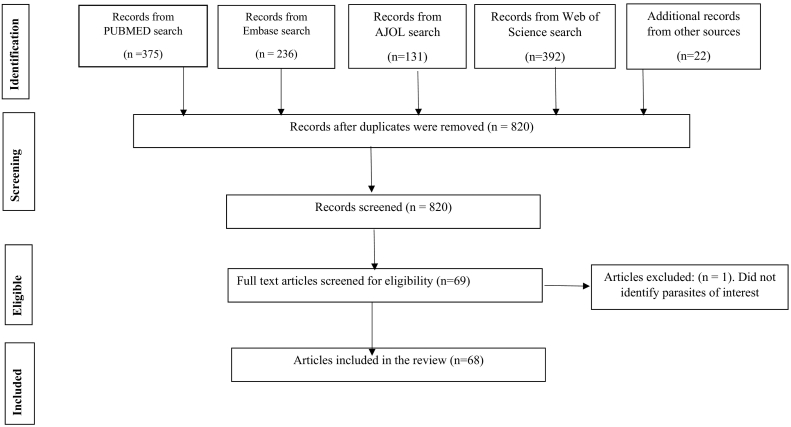
Diagram for a review on Protozoan parasites *Cryptosporidium*, *Giardia*, *Cyclospora cayetanensis* and *Entamoeba* in water and fresh produce 1990–January 2020.

The search was done in Medline via PubMed, Web of Science via the Web of Knowledge, Embase and African Journals online databases. A search phrase was developed based on Medline index terms and adapted to the other three databases (CM) (suppl2). The literature search was conducted on February 13, 2020. In addition, reference lists in the included articles were searched to capture possible additional publications. Majority of publications were excluded based on the title and abstract for not meeting the inclusion criteria. Independently, JS and FM read through all the retrieved articles, one-by-one and selected those that, reported studies on *Cryptosporidium*, *Giardia*, *Cyclospora* and *Entamoeba* conducted in any one of the African Countries. CM was responsible for quality assurance. In the third step, JS searched all additional relevant articles cited in the list of references of each of the initially selected articles. The selection of articles for inclusion from the search list was done based on the inclusion and exclusion criteria. The Preferred Reporting Items for Systematic Reviews and Meta-Analyses (PRISMA) flow diagram for this study is presented in Fig. 1.

### 2.4 Charting the results

Data extraction included basic information such as country, study site, author, publication year, study aims, sample type and sample size, diagnostic methods, number of positive samples, reported outcome measure such as prevalence or incidence, and incidental findings of other parasites. Prevalence was the major outcome measure and data has been presented according to parasite and sample type. In studies where prevalence was not given, it was estimated. Due to the heterogeneity of included studies, data was summarised and presented in tables without determining the overall average prevalence. Furthermore, incidental parasite isolations beyond the intended parasites were also reported.

## 3 Results

The results are presented in the following order; general findings (Section 3.1), prevalence and distribution of the four protozoan parasites (*Cryptosporidium* Section 3.2, *Giardia* Section 3.3, *C. cayetanensis* Section 3.4, *Entamoeba histolytica*/*dispar* Section 3.5) in water and sewage/effluent and, a combined prevalence and distribution of all four parasites in fresh produce (Section 3.6). Additionally, the prevalence of other parasites found in the various samples is presented (3.7, 3.8). As a number of studies did not provide prevalence estimates, these were calculated based on the absolute numbers provided and are presented as percentages.

### 3.1 General findings

**Fig. 2:**
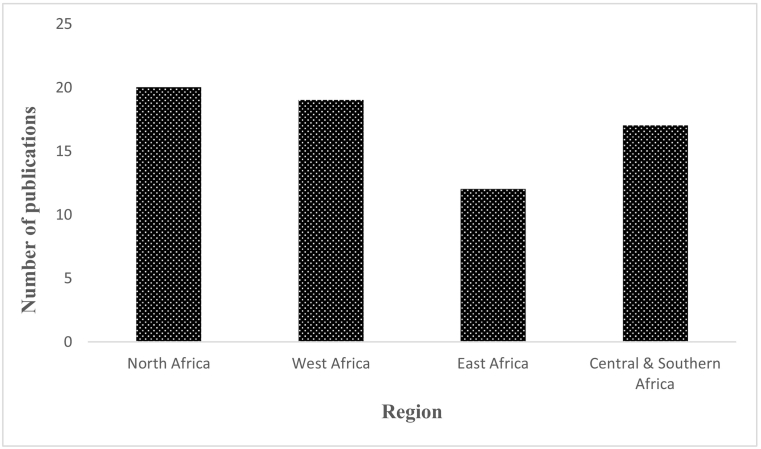
Number of publications on protozoan parasites in water and/or fresh produce according to region.

**Fig. 3:**
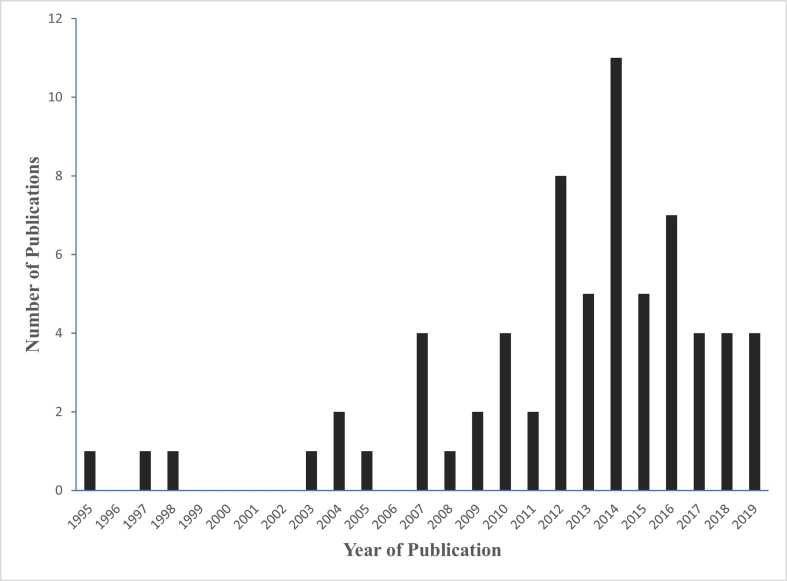
Number of publications on protozoan parasites in water and/or fresh produce in Africa.

A total of 1134 scientific articles were retrieved from the data bases, of which 46 were included in the review. Additional publications (22) which were captured from the reference lists of the 46 included studies were also included giving a total of 68 articles. All the reviewed articles were published from 1990 to January 2020. Of these, 62 were full-length articles, three conference papers (Samie and Mashau, 2012; Petersen et al., 2014; Medani et al., 2016) and three theses (Duhain, 2011; Markos, 2013; Sampson, 2015). The publications were distributed throughout Africa covering 17 countries (Algeria, Burkina Faso, Cameroon, Cote d'Ivore, Egypt, Ethiopia, Ghana, Libya, Kenya, Nigeria, Rwanda, South Africa, Sudan, Tunisia, Uganda, Zambia and Zimbabwe) with North African countries accounting for the majority of the articles (29.4%; 20/68) while east Africa accounted for 17.6% (12/68) (Fig. 2). Egypt had the highest number of publications (11) followed by Ethiopia, Ghana, Nigeria and South Africa which had eight publications each. There were more publications from the year 2012 onwards with a peak in 2014 (Fig. 3). The studies assessed the occurrence and/or prevalence of *Cryptosporidium* or *Giardia* or *Cyclospora* or *Entamoeba* or a combination of the parasites in fresh vegetables, fruits, surface water (rivers, lakes, dams, wells, ponds), waste water (treated effluents, untreated effluents) and tap water. All publications, except one (Ssemanda et al., 2018) reported positive results for any of the parasites under review, either as a single parasite or in combination with the other parasites. A total of 51 publications reported *Cryptosporidium*, while 47 reported *Giardia*, 23 *Entamoeba histolytica*/*dispar* and 15 reported *Cyclospora cayetanensis*. Helminths and other protozoan parasites and various bacterial forms were also isolated in the various water sources, wastewater and sludge.

Diagnostic methods used to identify the parasites varied per study. For *Cryptosporidium* and/or *C. cayetanensis*, various studies employed different diagnostic methods including modified Ziehl Neelsen, modified Bailengar, immunofluorescence, Auramine O-phenol, immunomagnetic separation and staining and EPA 1623. For *Giardia* and/or *Entamoeba*, wet smears (after a concentration method) with or without iodine stains, and Zinc Sulfate floatation were used to identify the cysts. For water samples, they were either filtered before concentration or directly concentrated before examination. A few studies (15) employed PCR in the analysis of the water.

**Fig. 4:**
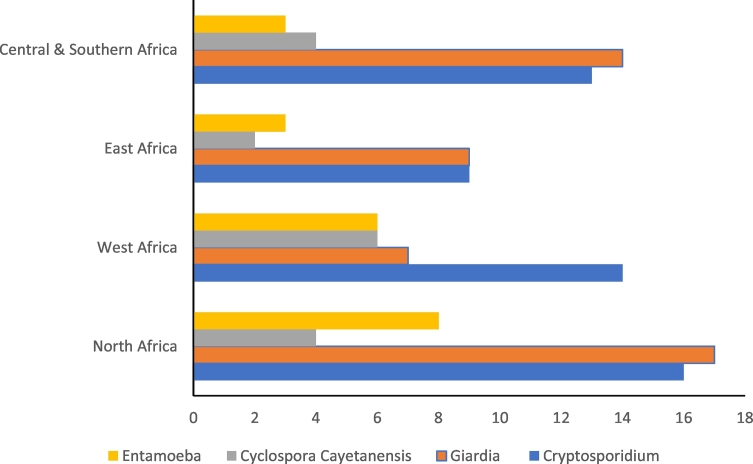
Number of publications on *Cryptosporidium*, *Giardia*, *Cyclospora cayetanensis* and *Entamoeba* in water and/or fresh produce in Africa according to region.

The highest number of articles reporting *Cryptosporidium* was from North Africa followed by West Africa and Central and Southern Africa (Fig. 4). Further, North Africa accounted for the highest number of studies reporting *Giardia* in various water bodies while East Africa had the least. East Africa also had the least number of publications reporting *C. cayetanensis* and *Entamoeba* (Fig. 4).

Fresh produce (fruits and vegetables) that are eaten raw varies from country to country. Carrots, onions, tomatoes, green pepper, lettuce, green pepper, parsley, watercress, tomato, cucumber and cabbage were the common vegetables reported to be eaten raw in the reviewed publications. Others, mostly from west African countries included green leaf (ugwu leaf, bitter leaf, sokoyokoto, igbagba), fluted pumpkin, waterleaf, morning glory solanum and curry (Alakpa et al., 2003; Chijioke et al., 2018). Fruits screened included mango, orange, lime, banana and cherry (Hassan et al., 2013; Tefera et al., 2014; Bekele et al., 2017; Chilo et al., 2018). Nineteen publications on contamination of fruits and vegetables were retrieved. Details of the parasite prevalence and distribution in various countries are described in Section 3.6.

### 3.2 Prevalence and distribution of *Cryptosporidium* in water and sewage/effluents

For purposes of this review, water samples were categorized into surface (if obtained from river, lakes, sea, pond or well), tap (if obtained from some form of conveyance such as a tap or hydraulic pump or borehole) or waste water when specified as such. Majority of the publications were on surface water (39/68). Table 1 summarises the distribution and reported estimates of *Cryptosporidium* in various water sources and sewage/effluents based on the specific African countries. Thirty-three of the 39 publications (85%) on various water sources reported occurrence of *Cryptosporidium*. The estimates are tabulated in Table 1. The reported prevalence of *Cryptosporidium* ranged from 6% (Sente et al., 2016) to over 58% (Grundlingh and De Wet, 2004; Ajeagah et al., 2010; Amenu et al., 2013) in surface water while the prevalence in tap water (sachet water was considered as tap water) ranged from 4% (Ndur et al., 2015) to 100% (Fikrie et al., 2008) (Table 1). *Cryptosporidium* oocysts were also recovered in wastewater, sludge and sewage (Table 1) with prevalence as high as 100% (Dungeni and Momba, 2010). The regional prevalence in various water sources and effluent/sewage was reported at 1–43% in North Africa,0.9–100% in West Africa, 7–100% in East Africa and 3–100% in Central and Southern Africa.

**Table 1:** Parasite distribution in positive water samples among various countries in Africa.

Parasite	Water sample type	Country reported	Prevalence range	References
*Cryptosporidium*	Surface water	Cameroon	25–100%	Ajeagah et al., 2007; Ajeagah et al., 2010; Chia et al., 2015; Nsoh et al., 2016
		Cote d'Ivore	28%	Koffi et al., 2014
		Egypt	3.1%	El-Shazly et al., 2007; Abd El-Salam, 2012; Khalifa et al., 2014; El-Kowrany et al., 2016
		Ethiopia	59–75%	Atnafu et al., 2012; Amenu et al., 2013
		Ghana	54–75%	Petersen et al., 2014; Sampson, 2015
		Kenya	7–8%	Kato et al., 2003; Muchiri et al., 2009
		Nigeria	100%	Uneke and Uneke, 2007
		South Africa	6–100%	Grundlingh and De Wet, 2004; Duhain, 2011
		Sudan	1–43%	Shanan et al., 2015; Medani et al., 2016
		Uganda	7%	Sente et al., 2016
		Zimbabwe	3–55%	Dalu et al., 2011; Mtapuri-Zinyowera et al., 2014
	Tap water	Egypt	28–36%	Hassan et al., 2012a; Sakran et al., 2017
		Ethiopia	21–100%	Fikrie et al., 2008; Atnafu et al., 2012
		Zambia	83%	Kelly et al., 1997
		Zimbabwe	22%	Dalu et al., 2011
		Ghana	9–63%	Kwakye-Nuako et al., 2007; Ndur et al., 2015; Tetteh-Quarcoo et al., 2016
		Egypt	7–36%	Youssef et al., 1998; Ali et al., 2004; Hamdy et al., 2019
		Uganda	40%	Sente et al., 2016
	Wastewater	South Africa	59–100%	Dungeni and Momba, 2010; Samie and Mashau, 2012
		Tunisia	25–35%	Khouja et al., 2010; Ben Ayed et al., 2012
	Sewage/sludge	South Africa	30%	Kfir et al., 1995
		Tunisia	20–42%	Khouja et al., 2010; Ben Ayed et al., 2012

### 3.3 Prevalence and distribution of *Giardia* in water and sewage/effluents

From all the articles captured in this review, *Giardia* was the second most common parasite after *Cryptosporidium*; with 47 out of 68 articles reporting the parasite. In publications reporting both *Cryptosporidium* and *Giardia*, *Giardia* appeared to have higher prevalence rates (Fig. 4). The prevalence in surface water varied from 2.4% (Khalifa et al., 2014) to 100% (Ajeagah et al., 2005, Ajeagah et al., 2010) while that in tap water ranged from 1% (Atnafu et al., 2012) to over 70% (Fikrie et al., 2008) (Table 2).

**Table 2:** Prevalence and distribution of *Giardia* in water and sewage/effluents among various African countries.

Parasite	Water sample type	Country reported	Prevalence range	References
*Giardia*	Surface water	Burkina Faso	5%	Kpoda et al., 2015
		Cameroon	4–100%	Ajeagah et al., 2005; Ajeagah et al., 2007; Ajeagah et al., 2010; Chia et al., 2015; Nsoh et al., 2016
		Cote d'Ivore	29%	Koffi et al., 2014
		Egypt	2–10%	El-Shazly et al., 2007; Abd El-Salam, 2012; Khalifa et al., 2014; El-Kowrany et al., 2016
		Ethiopia	65%	Amenu et al., 2013
		South Africa	23–100%	Grundlingh and De Wet, 2004; Duhain, 2011
		Sudan	71%	Medani et al., 2016
		Uganda	36%	Sente et al., 2016
		Zimbabwe	7–89%	Dalu et al., 2011; Mtapuri-Zinyowera et al., 2014
	Tap water	Egypt	6–8%	Ali et al., 2004; Sakran et al., 2017
		Ethiopia	1–73%	Fikrie et al., 2008; Atnafu et al., 2012
		Zimbabwe	0%	Dalu et al., 2011
		Sudan	14%	Mohamed et al., 2016
		Egypt	25%	Hamdy et al., 2019
		South Africa	25%	Dobrowsky et al., 2014
		Uganda	38%	Sente et al., 2016
		Ethiopia	30%	Markos, 2013
	Wastewater	Algeria	100%	Hamaidi-Chergui et al., 2019
		Cote d'Ivore	70%	Yapo et al., 2014
		South Africa	100%	Dungeni and Momba, 2010
		Tunisia	28–85%	Khouja et al., 2010; Ben Ayed et al., 2012
	Sewage/sludge	South Africa	33–50%	Kfir et al., 1995; Samie and Ntekele, 2014
		Tunisia	25–100%	Ben Ayed et al., 2009; Khouja et al., 2010; Ben Ayed et al., 2012

Articles on *Giardia* in wastewater, sludge/sewage were from Algeria, Cote d'Ivore, South Africa and Tunisia; the parasite estimates are indicated in Table 2. Prevalence ranged from 28% (Ben Ayed et al., 2012) to 100% of the tested sites and/or samples (Dungeni and Momba, 2010; Hamaidi-Chergui et al., 2019). The regional prevalence in various water sources and effluent/sewage ranged from 2 to 100% in North Africa, 0 to 100% in Central and Southern Africa, 5 to 70% in West Africa, and 1 to 73% in East Africa.

### 3.4 Prevalence and distribution of *Cyclospora cayetanensis* in water and sewage/effluents

Like *Cryptosporidium* and *Giardia*, *Cyclospora cayetanensis* was isolated from various water sources, wastewater and fresh produce. In surface water, the prevalence of *C. cayetanensis* ranged from 0.2% (El-Shazly et al., 2007) to 22% (2/9) (Dalu et al., 2011). In tap water, the prevalence was low for all studies reporting the parasite while in wastewater, Ben Ayed et al. (2012) reported the highest prevalence of 45% (see Table 3). The same article by Ben Ayed et al. (2012) further reported a *C. cayetanensis* prevalence of 58% in sludge (Table 3). The regional prevalence in various water sources and effluent/sewage was 0.2–58% in North Africa, 0–22% in Central and Southern Africa and 5–7% in West Africa.

**Table 3:** Prevalence and distribution of *Cyclospora cayetanensis* in water and sewage/effluents among African countries.

Parasite	Water sample type	Country reported	Prevalence range	References
*Cyclospora cayetanensis*	Surface water	Egypt	0.2–4%	El-Shazly et al., 2007; Khalifa et al., 2014
		Zimbabwe	10–22%	Dalu et al., 2011; Mtapuri-Zinyowera et al., 2014
	Tap water	Ghana	5–7%	Ndur et al., 2015; Tetteh-Quarcoo et al., 2016
		Zimbabwe	0%	Dalu et al., 2011
	Wastewater	South Africa	15%	Samie and Mashau, 2012
		Tunisia	45%	Ben Ayed et al., 2012
	Sludge	Tunisia	58%	Ben Ayed et al., 2012

### 3.5 Prevalence and distribution of *Entamoeba histolytica*/*dispar* in water and sewage/effluents

Most studies did not apply molecular methods to distinguish *E. histolytica* from *E. dispar* and results were therefore reported as positive for *E. histolytica*/*dispar*. Similar to the other parasites discussed above, *Entamoeba histolytica*/*dispar* was reported in various water bodies and sources (Table 4) and from all the four categorized regions (North, West, East and Central and Southern Africa) with prevalence ranging from 8 to 36% in West Africa, 0.3 to 100% in North Africa, and 0 to 78% in Southern Africa. In surface water, the prevalence ranged from 1% (El-Shazly et al., 2007) to 78% (Mtapuri-Zinyowera et al., 2014). No publication reported *Entamoeba* spp. oocysts in tap water. In wastewater, two articles reported a prevalence of 100% (Khouja et al., 2010; Hamaidi-Chergui et al., 2019) and Ben Ayed et al. (2009) also reported a 100% prevalence in sludge.

**Table 4:** Prevalence and distribution of *Entamoeba* in water and sewage/effluents among various African countries.

Parasite	Water sample type	Country reported	Prevalence range	References
*E. histolytica*	Surface water	Burkina Faso	8%	Kpoda et al., 2015
		Cameroon	16%	Nsoh et al., 2016
		Cote d'Ivore	35%	Koffi et al., 2014
		Egypt	1–3%	El-Shazly et al., 2007; Khalifa et al., 2014
		Nigeria	36%	Bishop and Inabo, 2015
		Sudan	0.3%	Shanan et al., 2015
		Zimbabwe	13–78%	Dalu et al., 2011; Mtapuri-Zinyowera et al., 2014
	Tap	Zimbabwe	0%	Dalu et al., 2011
	Wastewater	Algeria	100%	Khouja et al., 2010; Hamaidi-Chergui et al., 2019
	Sewage/sludge	Tunisia	50–100%	Ben Ayed et al., 2009; Khouja et al., 2010

### 3.6 Prevalence and distribution of *Cryptosporidium*, *Giardia*, *Cyclospora cayetanensis* and *Entamoeba* in fresh produce

Nineteen publications out of the 68 publications reported contamination of fruits and vegetables with the subject parasites. The prevalence of *Cryptosporidium* on fresh produce (fruits and vegetables) ranged from 0.8% (Tchounga et al., 2017) to over 80% (Saaed and Ongerth, 2019). Most publications that reported the parasites in vegetables/fruits were from West Africa (8/12), this review did not capture any article from the Central and Southern African region (Table 5). The prevalence in East, West and North Africa were reported at 5–13%, 0.8–75% and 29–81% respectively.

**Table 5:** Prevalence and distribution of *Cryptosporidium*, *Giardia*, *Cyclospora cayetanensis* and *Entamoeba* in fresh produce among various African countries.

Parasite	Sample type	Country reported	Prevalence range	References
*Cryptosporidium*	Vegetables	Egypt	29%	El Said Said, 2012
		Ethiopia^a^	5–13%	Tefera et al., 2014; Bekele et al., 2017
		Ghana^b^	30%	Ayeh-Kumi et al., 2014
		Ghana	11–75%	Duedu et al., 2014; Petersen et al., 2014; Sampson, 2015; Kudah et al., 2018
		Libya	81%	Saaed and Ongerth, 2019
		Nigeria	0.8–35%	Maikai et al., 2013; Hassan et al., 2013; Tchounga et al., 2017
*Giardia*	Vegetables	Egypt	6–8%	El Said Said, 2012; Hassan et al. (2012b)
		Ethiopia^a^	8–28%	Tefera et al., 2014; Bekele et al., 2017; Chilo et al., 2018
		Ghana	13%	Duedu et al., 2014
		Libya	46–84%	Abougrain et al., 2010; Saaed and Ongerth, 2019
		Nigeria	2–99%	Amaechi et al., 2016; Tchounga et al., 2017; Chijioke et al., 2018
	Vegetables	Sudan	3%	Mohamed et al., 2016
*Cyclospora cayetanensis*	Vegetables	Egypt	21%	El Said Said, 2012
		Ethiopia^a^	5–7%	Tefera et al., 2014; Bekele et al., 2017
		Ghana	23%	Ayeh-Kumi et al., 2014
		Ghana	12%	Duedu et al., 2014
*Entamoeba histolytica*	Vegetables	Burkina Faso	20%	Kpoda et al., 2015
		Egypt	7%	Hassan et al. (2012b)
		Ethiopia^a^	5–24%	Tefera et al., 2014; Bekele et al., 2017; Chilo et al., 2018
		Ghana	26%	Duedu et al., 2014
		Nigeria	6–99%	Hassan et al., 2013; Amaechi et al., 2016; Tchounga et al., 2017; Chijioke et al., 2018
		Sudan	6%	Mohamed et al., 2016

a Vegetables and fruits.

b Tiger nuts.

Publications reporting *Giardia* cysts from fruits and vegetables in various countries are indicated in Table 5. *Giardia* cysts were identified in over 50% of the studies (73.7%; 14/19). Prevalence rates ranged from 2% (3/150) (Amaechi et al., 2016) to over 99% (159/160) (Chijioke et al., 2018). The regional prevalence in North, West and East Africa ranged from 3 to 84%, 2 to 99% and 8 to 28% respectively. Similar studies from central and southern Africa appear to be lacking as no articles on the subject matter were captured in the search.

A few (five) publications reported *C. cayetanensis* in fruits and vegetables with relatively lower prevalence rates (range: 5%–23%) (Table 5).

*Entamoeba histolytica*/*dispar* was also isolated from fresh fruits and vegetables (Table 5) with varying prevalence ranging from 3% (Hassan et al., 2013) to 99% (Chijioke et al., 2018). West Africa accounted for most of the articles (Table 5).

### 3.7 Prevalence and distribution of other protozoan parasites in water, sewage/effluents & fresh produce

The subject protozoan parasites in this review were *Cryptosporidium*, *Giardia*, *Cyclospora cayetanensis* and *Entamoeba*. The reviewed articles also reported other protozoa as tabulated in Table 6 with varying prevalence rates. In surface water, the following protozoa were reported: *Toxoplasma gondii*, *Sarcocystis* spp., *Isospora belli*, *Balantidium coli*, *Blastocystis hominis*, *Acanthamoeba*, *Entamoeba coli*, *Trichomonas vaginalis* and *Chilomastix mesnilli*; in wastewater. *Isospora belli* and *Eimeria* spp. were reported while in tap water, only *Sarcocystis* was reported (Table 6). *Balantidium coli*, *T. gondii* and *I. belli* were also reported in vegetables (Table 6).

**Table 6:** Prevalence and distribution of other protozoan parasites reported in water, sewage/effluents & fresh produce in African countries.

Parasite	Sample type	Country reported	Prevalence range	References
*Toxoplasma gondii*	Surface water	Sudan	0.7–81%	Shanan et al., 2015; Medani et al., 2016
*Blastocystis hominis*	Surface water	Cameroon	4%	Nsoh et al., 2016
		Egypt	1%	El-Shazly et al., 2007
		Sudan	0.2%	Shanan et al., 2015
*Acanthamoeba*	Surface water	Egypt	73%	Sakran et al., 2017
		Sudan	1%	Shanan et al., 2015
*Trichomonas vaginalis*	Surface water	Sudan	0.2%	Shanan et al., 2015
*Sarcocystis*	Surface water	Cameroon	7%	Nsoh et al., 2016
*Isospora belli*	Surface water	Cameroon	4%	Nsoh et al., 2016
		Egypt	0.5%	El-Shazly et al., 2007
*Balantidium coli*	Surface water	Cameroon	4%	Nsoh et al., 2016
*Chilomastix mesnilli*	Surface water	Egypt	0.1%	El-Shazly et al., 2007
*Entamoeba coli*	Surface water	Burkina Faso	6%	Kpoda et al., 2015
*Sarcocystis*	Tap water (sachets)	Ghana	67%	Kwakye-Nuako et al., 2007
*I. belli*	Wastewater	South Africa	18%	Samie and Mashau, 2012
*Eimeria* spp.	Wastewater	Tunisia	81%	Ben Ayed et al., 2012
	Sludge	Tunisia	58%	Ben Ayed et al., 2012
*Balantidium coli*	Vegetables	Ghana	14%	Kudah et al., 2018
	Vegetables	Nigeria	3%	Amaechi et al., 2016
*T. gondii*	Vegetables	Nigeria	3%	Tchounga et al., 2017
*I. belli*	Vegetables	Nigeria	2%	Hassan et al., 2013

### 3.8 Helminth parasites and other pathogens in water, sewage/effluents & fresh produce

Various helminth species were reported in all water types and in wastewater (Table 7). However, not all articles provided estimates for the specific parasite species, making it difficult to make comparisons. In surface water, *Ascaris lumbricoides*, *Strongyloides stercoralis*, *Ancylostoma duodenale*, *Enterobius vermicularis*, hookworm; *Taenia* spp., *Hymenolepis nana* were reported (Dalu et al., 2011; Bishop and Inabo, 2015; Mtapuri-Zinyowera et al., 2014; Kpoda et al., 2015; Ndur et al., 2015; Mohamed et al., 2016). In wastewater, *Ascaris*, *Trichuris* spp., *H. nana*, *H. diminuta*, *E. vermicularis*, *Taenia* spp. and *Toxocara* spp. were reported (Ben Ayed et al., 2009; Khouja et al., 2010; Hamaidi-Chergui et al., 2019).

**Table 7:** Prevalence and distribution of helminth parasites reported in water, sewage/effluents & fresh produce in African countries.

Helminths	Sample type	Country reported	Prevalence range	References
*Ascaris lumbricoides*	Surface water	Burkina Faso	8%	Kpoda et al., 2015
	Tap (sachet)	Ghana	10%	Ndur et al., 2015
	Sprinkling water	Sudan	29%	Mohamed et al., 2016
	Tap, stored	Zimbabwe	22%	Dalu et al., 2011
	Wastewater	Algeria	100%	Hamaidi-Chergui et al., 2019
		Tunisia	71%	Khouja et al., 2010
	Sewage	Tunisia	100%	Ben Ayed et al., 2009
	Vegetables	Egypt	20%	El Said Said, 2012
	Vegetables/fruits	Ethiopia	7–32%	Tefera et al., 2014; Bekele et al., 2017; Chilo et al., 2018
	Vegetables	Libya	68%	Abougrain et al., 2010
		Nigeria	19–100%	Hassan et al., 2013; Amaechi et al., 2016; Tchounga et al., 2017; Chijioke et al., 2018
		Sudan	3%	Mohamed et al., 2016
*Ancylostoma duodenale* (hookworm)	Surface water	Burkina Faso	31%	Kpoda et al., 2015
	Sprinkling water	Sudan	6%	Mohamed et al., 2016
	Tap, stored	Zimbabwe	44%	Dalu et al., 2011
	Vegetables	Burkina Faso	20%	Kpoda et al., 2015
	Tiger nuts	Ghana	25%	Ayeh-Kumi et al., 2014
	Vegetables	Ghana	13%	Duedu et al., 2014
		Sudan	6%	Mohamed et al., 2016
		Nigeria	12%	Tchounga et al., 2017
*Strongyloides stercolaris*	Surface water	Burkina Faso	3%	Kpoda et al., 2015
		Nigeria	7%	Bishop and Inabo, 2015
	Tap (sachet)	Ghana	5%	Ndur et al., 2015
	Sprinkling water	Sudan	43%	Mohamed et al., 2016
	Tap, stored	Zimbabwe	11%	Dalu et al., 2011
	Vegetables/fruits	Ethiopia	22%	Tefera et al., 2014
	Tiger nuts	Ghana	22%	Ayeh-Kumi et al., 2014
	Vegetables	Ghana	36–43%	Duedu et al., 2014; Kudah et al., 2018
		Nigeria	2–20%%	Hassan et al., 2013; Amaechi et al., 2016; Tchounga et al., 2017
		Sudan	9%	Mohamed et al., 2016
*Enterobius vermicularis*	Surface	Nigeria	3%	Bishop and Inabo, 2015
	Wastewater &sludge	Tunisia	37%	Khouja et al., 2010
	Sewage	Tunisia	100%	Ben Ayed et al., 2009
	Vegetables	Ghana	1%	Duedu et al., 2014
		Nigeria	12–89%	Amaechi et al., 2016; Chijioke et al., 2018
*Trichuris trichiura*	Wastewater	Algeria	100%	Hamaidi-Chergui et al., 2019
	Vegetables	Ghana	2%	Duedu et al., 2014
		Nigeria	6–89%	Hassan et al., 2013; Amaechi et al., 2016; Chijioke et al., 2018
		Sudan	3%	Mohamed et al., 2016
*Schistosoma* spp.	Tap, stored	Zimbabwe	22%	Dalu et al., 2011
	Vegetables	Nigeria	44%	Chijioke et al., 2018
*Taenia* spp.	Wastewater	Algeria	100%	Hamaidi-Chergui et al., 2019
	Wastewater & sludge	Tunisia	14%	Khouja et al., 2010
	Sewage	Tunisia	47%	Ben Ayed et al., 2009
	Vegetables	Libya	22%	Abougrain et al., 2010
		Nigeria	2–67%	Hassan et al., 2013; Chijioke et al., 2018
*Toxocara* spp.	Wastewater	Algeria	100%	Hamaidi-Chergui et al., 2019
	Vegetables/fruits	Ethiopia	15–16%	Tefera et al., 2014; Bekele et al., 2017
	Vegetables	Libya	16–26	Abougrain et al., 2010
*Hymenolepis nana*	Wastewater	Algeria	100%	Hamaidi-Chergui et al., 2019
	Wastewater & sludge	Tunisia	14%	Khouja et al., 2010
	Sewage	Tunisia	18%	Ben Ayed et al., 2009
	Vegetables	Egypt	3%	El Said Said, 2012
	Vegetables/fruits	Ethiopia	8–16%	Tefera et al., 2014; Bekele et al., 2017; Chilo et al., 2018
*Hymenolepis diminuta*	Wastewater	Algeria	100%	Hamaidi-Chergui et al., 2019
	Vegetables/fruits	Ethiopia	1–8%	Tefera et al., 2014; Bekele et al., 2017
*Fasciola* spp.	Vegetables	Ghana	0.2–7%	Duedu et al., 2014; Kudah et al., 2018
		Nigeria	8–78%	Amaechi et al., 2016; Tchounga et al., 2017; Chijioke et al., 2018

From fresh produce, nematodes (*A. lumbricoides*, *S. stercolaris*, *Ancylostoma duodenale*, *E. vermicularis*, hookworm) were commonly reported in most publications cestodes (*Taenia* spp., *Hymenolepis nana*, *H. diminuta*) and trematodes (*Fasciola* spp., *Schistosoma mansoni*) were also reported (Table 7).

Bacterial forms were also reported in surface water by Amenu et al. (2013); Koffi et al. (2014); Khalifa et al. (2014) in Ethiopia, Cote d'Ivore and Egypt respectively; tap water in Zimbabwe, South Africa and Ghana (Dalu et al., 2011; Dobrowsky et al., 2014; Tetteh-Quarcoo et al., 2016) and waste water in Cote d'Ivore (Yapo et al., 2014). In vegetables, bacteria were reported from studies from Ghana and Ethiopia (Ayeh-Kumi et al., 2014; Chilo et al., 2018). One publication from Egypt reported viruses in tap water (Ali et al., 2004).

## 4 Discussion

This review has brought out important findings indicating the widespread contamination of African water bodies, tap water and fresh produce with protozoan parasites *Cryptosporidium*, *Giardia*, *Cyclospora* and *Entamoeba*. The review has also revealed the presence of the protozoa and other parasites in sewage and treated effluents. A significant number of articles have been published across the African continent on *Cryptosporidium* and *Giardia* in water, fruits and vegetables especially after the year 2010 (Fig. 3). There is, however, lack of uniformity in the design, determination of sample size and reporting of findings making it difficult to compare the various studies.

The review has further revealed increasing research on the waterborne protozoan parasites in various water sources and fresh produce over time. Publications were distributed throughout Africa with North African countries accounting for the majority of the articles while East Africa had the least number (Fig. 2). However, despite the clustering in the northern region, some countries produced more publications than others, for example, Egypt had 11 publications while there was only one from Algeria. The status was similar for east Africa where there is limited information in Uganda and Kenya compared to Ethiopia. There is need for more countrywide research in the various parts of the continent to appreciate the burden of the subject parasites in Africa.

Studies in surface water revealed widespread contamination with *Cryptosporidium* and *Giardia*, information which corroborates with studies in developed countries (Bouzid et al., 2008; Karanis et al., 2007). For *Cryptosporidium*, prevalence of over 50% (Grundlingh and De Wet, 2004; Amenu et al., 2013) were reported in most studies while *Giardia* prevalence was as high as 100% in some of the water samples tested (Ajeagah et al., 2005, Ajeagah et al., 2010). Despite the variations in sample size, it is still clear that surface African surface waters are contaminated with the two important waterborne parasites. Tap water was equally contaminated, including packaged water (in sachets) (Ndur et al., 2015). Even though no major waterborne outbreak of diarrhoeal disease linked to protozoa contamination of water has been reported in Africa, the two parasites have previously been associated with waterborne outbreaks in the USA and other parts of the world (MacKenzie et al., 1994; Karanis et al., 2007). The high burden of *Cryptosporidium* and *Giardia* demonstrated in the reviewed studies in surface water, untreated and treated effluents provide evidence necessary to carry out causation studies in Africa. It is possible that contamination of water bodies (some of which are sources of water for many households) and tap water by these parasites could be responsible for some of the diarrhoea disease in affected countries. Future human studies reporting *Cryptosporidium* and/or *Giardia* infections should endeavor to establish sources of infections as this will assist in coming up with targeted intervention measures.

The review also highlighted a high level of parasite contamination in wastewater (treated and untreated) and sewage/sludge. Wastewater as well as treated effluents were variably contaminated with the parasites under study. Wastewater (treated and untreated) has been reported to be increasingly being used in agriculture with the majority in untreated form in developing countries due to scarcity of water (Scott et al., 2010; Dickin et al., 2016). The levels of contamination exhibited in this review therefore raises concern. In places where wastewater may be used for vegetable cultivation, such contamination as seen in the reviewed publications poses a public health risk. It is important that such acts, where practiced, are discouraged and safe water should be provided to communities to safeguard their health. In the current review, 13 out of the 19 studies and 14 of the 19 studies on parasite contamination in vegetables and/or fruits that are normally eaten raw reported presence of *Cryptosporidium* and *Giardia* parasites, respectively. *Cyclospora* cayetanensis and *E. histolytica* were also detected (Table 5). With the known clinical consequences of human infection with these parasites especially in immunocompromised individuals (Supplementary Table 1) and the zoonotic potential of the parasites, it is important that people are sensitized about the risks of consuming raw or undercooked foods. Health education could therefore assist in preventing infections. Fresh produce can be contaminated with enteric pathogens throughout the process of planting to consumption. It is therefore, important that strict hygiene measures are advocated for in the production process as well as at household level to prevent human infections. Several publications also reported presence of other pathogens including helminths, other protozoa and bacteria in water, sewage and effluents and fruits and vegetables (Table 6, Table 7). It is evident that there is widespread helminths contamination especially in surface water and vegetables.

With the current evidence, it is important that research linking diarrhoea disease with contamination of water bodies/sources or fresh produce with protozoan parasites be carried out in Africa. This will provide evidence-based data on the various possible causes and sources of diarrhoea disease especially in children and immunocompromised individuals in disadvantaged communities. Furthermore, only 17 out of the 54 African countries have conducted studies on these parasites; more studies are necessary in other countries to provide conclusive evidence on the exact burden of the four protozoan parasites in water sources, sewage/effluents and fresh produce.

The limitation of this review is the possibility of failing to capture publications that are not indexed in the searched data bases. Further, only four databases were searched. However, we believe most were obtained from the reference lists of the reviewed publications and these sufficiently give a picture of the status of the target parasites in Africa. Secondly, some studies did not strictly follow the STROBE guidelines making it impossible to extract uniform data from the articles. Another limitation is that most studies did not perform molecular tests to distinguish *E. histolytica* and *E dispar*. The prevalence of *E. histolytica* may therefore be overestimated.

## 5 Conclusions and future perspectives

In many African communities, particularly those in rural areas, individuals have limited access to adequate and safe household water. This coupled with inadequate or lack of water treatment, poor hygiene practices, and lack of awareness and education programmes, significantly contributes to predisposition of many such communities to parasitic infections. With population growth in the midst of inadequate infrastructure, sanitary facilities and lack of a systematic way of determining the prevalence *Cryptosporidium*, *Giardia*, *Cyclospora* and *Entamoeba histolytica*/*dispar* from food and water sources, parasitism in humans in the African setting will continue to be a challenge. The findings in this review echo this. Despite the importance and popularity of swimming pools particularly in hotter months of year, reports of protozoan parasites in these waters poses a public health risk to users. Further, swimming in contaminated water bodies, a common practice in rural settings, adds to the public health risk to users of these facilities. Periodic screening for these parasites in treated and untreated raw water particularly at household level and provision of adequate safe drinking water is advocated for. This should be supported by education and awareness programmes on the importance of using clean and safe water at household level including washing of fresh produce. Active and passive surveillance should also be conducted, and efforts should be made to minimise dissemination of oocysts and cysts in the farming environment and via human waste management. To achieve meaningful outcomes, it is also important to note that diseases that may result from infection with the discussed parasites cannot be managed with one method alone; they require an integrated and one health approach.

The following are the supplementary data related to this article.

Supplementary Table 1 Summarised overview of *Cryptosporidium*, *Giardia*, *Cyclospora cayetanensis* and *Entamoeba*. Supplementary Table 1

Supplementary material Image 1

## Declaration of competing interest

The authors declare that they have no known competing financial interests or personal relationships that could have appeared to influence the work reported in this paper.
