# Supplementary material for: Food and waterborne protozoan parasites: The African perspective
Source: Food Waterborne Parasitol. 2020 Sep 9;20:e00088. doi: 10.1016/j.fawpar.2020.e00088 (PMC7502820; doi:10.1016/j.fawpar.2020.e00088)
Supplement: Supplementary Table 1 — Summarised overview of Cryptosporidium, Giardia, Cyclospora cayetanensis and Entamoeba. [file mmc1.docx]

**Supplementary table 1: Summarised overview of *Cryptosporidium,* *Giardia*, *Cyclospora cayetanensis* and *Entamoeba***

| **Parasite name** | **Transmission** | **Lifecycle** | **Clinical signs** | **Treatment** | **References** |
| --- | --- | --- | --- | --- | --- |
| *Cryptosporidium* spp. | Faecal-oral route, ingestion of infective oocysts | Has both asexual and sexual stages, developmental stages include oocysts, sporozoites, meronts (type I & II) and gametocytes (microgametocytes & macrogametocytes). Meronts produced by asexual reproduction of sporozoites, latter differentiate into gametocytes. Sexual reproduction produces infective sporulated oocysts, excreted in the faeces | Profusely watery diarrhoea, nausea, myalgia, weakness, malaise, headache, anorexia, abdominal pain and mild fever  Infection is self-limiting in immunocompetent individuals  Possible extra-intestinal infections affecting biliary tract, liver, stomach, lungs and pancreas | Nitazoxadine, more so in immunocompromised individuals, supportive therapy | Current & Garcia 1991; Lopez-Velez et al., 1995; Ramirez et al., 2004; Cacciò & Chalmers, 2016. |
| *Giardia duodenalis* | Oral-faceal route, ingestion of cysts | Asexual reproduction, two developmental stages (cyst & trophozoite).  Cyst excyst in small intestine release trophozoites which colonize and attach to sub-mucosal epithelial lining and reproduce | Asymptomatic, symptomatic cases present with acute foul-smelling diarrhoea, malabsorption, weight loss, cognitive impairment in children | Metronidazole | Farthing, 1999; Berkman et al., 2002; Einarsson et al., 2016; Certad et al., 2017; Squire and Ryan, 2017. |
| *Cyclospora cayetanensis* | Faecal-oral route, ingestion of sporulated oocysts | Asexual & sexual stages, developmental stages include oocysts, sporozoites, meronts and gametocytes.  Fertilisation produces unsporulated oocyst. Once excreted, oocyst sporulates in 5-7 days to become infective. Once ingested, cyst excyst, release sporozoites | Mostly asymptomatic; Nausea, headache, loss of appetite, abdominal cramps, profuse, watery diarrhoea occur in symptomatic patients.  Extra-intestinal complications in immune-compromised individuals | Trimethoprim/sulfamethoxazole is the treatment of choice, ciprofloxacin and nitazoxanide are proposed as alternatives | Ortega et al., 1994; Connor et al., 1999;  Eberhard et al., 2001, Ortega and Sanchez et al., 2010; Bednarska et al., 2015; Gomez Martínez et al., 2016). |
| *Entamoeba* | Faecal-oral route, ingestion of the cyst; sexual transmission can also occur | Asexual reproduction, two developmental stages (infective cyst and the proliferative trophozoite). Cysts excyst in small intestine, transform to trophozoites; trophozoites migrate to the large intestine, multiply and invade intestines, spread to other organs (e.g. liver) haematogenously. Cysts are excreted in faeces | Asymptomatic in approximately 90% of patients. Symptomatic patients present with acute colitis; invasive amoebiasis present with amoebic liver abscess | 5-Nitoimidazoles (metronidazole) are recommended for treatment | Salit et al., 2009; Hung et al., 2012, Mi-Chi et al., 2016; Carrero et al. 2020. |

**References**

Bednarska, M., Bajer, A., Welc-Falęciak, R., Pawełas, A., 2015. *Cyclospora cayetanensis* infection in transplant traveller: a case report of outbreak Parasites Vectors, 8, 411, 10.1186/s13071-015-1026-8.

Berkman, D.S., Lescano, A.G., Gilman, R.H., Lopez, S.L.L., Black, M.M., 2002. Effects of stunting, diarrhoeal disease, and parasitic infection during infancy on cognition in late childhood: a follow-up study Lancet, 359, 564-571. 10.1016/s0140-6736(02)07744-9.

Certad, G., Viscogliosi, E., Chabé, M., Cacciò, S.M., 2017. Pathogenic mechanisms of *Cryptosporidium* and *Giardia.* Trends in Parasitol, 33, 561-576. 10.1016/j.pt.2017.02.006

Connor, B.A., Reidy, J., Soave, R., 1999. Cyclosporiasis: clinical and histopathologic correlates. Clinical Infect. Dis., 28, 1216-1222. DOI: 1058–4838/99/2806–0006$03.00.

Current, W.L., Garcia L.S., 1991. Cryptosporidiosis. Clin. Microbiol. Rev., 4, 325-358. 10.1128/cmr.4.3.325.

Eberhard, M.L., Njenga, M.N., DaSilva, A.J., Owino, D., Nace, E.K., Won, K.Y., et al. 2001. A survey for *Cyclospora* spp. in Kenyan primates, with some notes on its biology J. Parasitol., 87, 1394-1397. 10.1645/00223395(2001)087[1394:ASFCSI]2.0.CO.2

Farthing, M.J.G., 1999. Giardiasis. In H.M. Gilles (Ed.), Protozoal Diseases, Oxford University Press, New York, USA, pp. 562-585

Gomez Martinez, E., Figuera, L., Guilarte, DelV., Simoni, Z., Tulio Diaz, M., Berrizbeitia, M., et al. 2016. First report of *Cyclospora cayetanensis* in an indigenous Kariña a community in Sucre state, Venezuela. Boletín de Malariología y Salud Ambiental, 56, 19-29. (*Abstract*)

Hung, C.C., Chang, S.Y., Ji D.D., 2012. *Entamoeba histolytica* infection in men *Entamoeba* who have sex with men The Lancet Infect. Dis., 12, 729-736. 10.1016/S1473-3099(12)70147-0

López-Vélez, R., Tarazona, R., Camacho, A.G., Gomez-Mampaso, E., Guerrero, A., Moreira, V., et al. Intestinal and extraintestinal cryptosporidiosis in AIDS patients. Eur. J. Clin. Microbiol. Infect. Dis., 14, 677-681, 10.1007/BF01690873.

Mi-Chi, F., Yoshida, H., Hamano, S., 2016. encystation: new targets to prevent the transmission of amoebiasis. PLoS Pathog., 12, article e1005845.doi:10.1371/journal.ppat.1005845.

Murray, C.J., Richards, M.A., Newton, J.N., Fenton, K. A., Anderson, H.R., Atkinson, C., et al. 2013. UK health performance: findings of the global burden of disease study 2010. Lancet, 381, 997-1020. 10.1016/S0140-6736(13)60355-4.

Ortega, Y.R., Sanchez, R., 2010. Update on *Cyclospora cayetanensis*, a food-borne and waterborne parasite. Clin. Microbiol. Rev., 23, 218-234. 10.1128/CMR.00026-09.

Ortega, Y.R., Gilman, R.H., Sterling, C.R., 1994. A new coccidian parasite (Apicomplexa: Eimeriidae) from humans. J. Parasitol., 80, 625-629. 10.2307/3283201. <https://www.jstor.org/stable/3283201>.

Ramirez, N.E., Ward, L.A., Sreevatsan, S., 2004. A review of the biology and epidemiology of cryptosporidiosis in humans and animals. Microbes Infect., 6, 773-785. Doi:10.1016/j.micinf.2004.02.021.

Squire, S.A., Ryan, U., 2017. *Cryptosporidium* and *Giardia* in Africa: current and future challenges. Parasites Vectors, 10, 195. Doi:10.1186/s13071-017-2111-y.

Cacciò, S.M., Chalmers, R.M., 2016. Human cryptosporidiosis in Europe. Clin Microbiol Infect. 22, 471-480. doi:10.1016/j.cmi.2016.04.021.

López-Vélez, R., Tarazona, R., Garcia Camacho, A., Gomez-Mampaso, E., Guerrero, A., Moreira, V., et al. 1995. Intestinal and extraintestinal cryptosporidiosis in AIDS patients. Eur J Clin Microbiol Infect Dis. 14, 677-681. doi:10.1007/BF01690873.

Einarsson, E., Ma’ayeh, S., Sva¨rd, S.G., 2016. An up-date on *Giardia* and giardiasis. Curr Opin Microbiol. 34,47–52. <http://dx.doi.org/10.1016/j.mib.2016.07.019>.

Salit, I.E., Khairnar, K., Gough, K., Pillai, D.R., 2009. A possible cluster of sexually transmitted *Entamoeba* *histolytica*: Genetic analysis of a highly virulent strain. Clin Infect Dis. 49, 346–353. <https://doi.org/10.1086/600298>.

Carrero, J.C., Reyes-López, M., Serrano-Luna, J, Shibayama, M., Unzueta, J., Nidia León-Sicairos, N., et al. 2020. Intestinal amoebiasis: 160 years of its first detection and still remains as a health problem in developing countries. Int J Med Microbiol. 310, 151358. doi:10.1016/j.ijmm.2019.151358.
