# Supplementary material for: Food and waterborne protozoan parasites: The African perspective
Source: Food Waterborne Parasitol. 2020 Sep 9;20:e00088. doi: 10.1016/j.fawpar.2020.e00088 (PMC7502820; doi:10.1016/j.fawpar.2020.e00088)
Supplement: Supplementary file 2 — Supplementary material [file mmc2.docx]

**Search Phrase**

The search word was developed using indexed terms in the Mesh database. The search word was open on subjects (humans, animals, and environment) in order not to miss any publications especially those which are combined. The search term was as follows;

(Cryptosporidium OR cryptosporidios* OR ‘Giardia lamblia’ OR ‘Giardia intestinalis’ OR ‘Giardia duodenalis’ OR ‘Giardia intestinalis’ OR Giardia* OR Lamblia* OR ‘Cyclospora cayetanensis’ OR Cyclospora OR Entamoebias* OR ‘Entamoeba histolytica’ OR ‘Amebic Dysentery’ OR ‘Amebic Dysenteries’ OR Amebias* OR Amoebias* OR ‘Amoebic Colitis’ OR ‘Amoebic Colitides’ OR ‘Amebic Colitis’ OR ‘Amoebic Dysentery’ OR ‘Amoebic Dysenteries’) AND (Epidemics OR frequency OR surveillance OR morbidity OR occurrence OR outbreaks OR prevalence OR endemics OR incidence OR cases) AND (Algeria OR Angola OR Benin OR Botswana OR Burkina Faso OR Burundi OR Cabo Verde OR Cameroon OR Central African Republic (CAR) OR Chad OR Comoros OR Congo, Democratic Republic of the OR Congo, Republic of the OR Cote d'Ivoire OR Djibouti OR Egypt OR Equatorial Guinea OR Eritrea OR Eswatini (formerly Swaziland) OR Ethiopia OR Gabon OR Gambia OR Ghana OR Guinea OR Guinea-Bissau OR Kenya OR Lesotho OR Liberia OR Libya OR Madagascar OR Malawi OR Mali OR Mauritania OR Mauritius OR Morocco OR Mozambique OR Namibia OR Niger OR Nigeria OR Rwanda OR Sao Tome and Principe OR Senegal OR Seychelles OR Sierra Leone OR Somalia OR South Africa OR South Sudan OR Sudan OR Tanzania OR Togo OR Tunisia OR Uganda OR Zambia OR Zimbabwe) AND (Transmission OR ‘risk factor’ OR exposure OR sporadic OR risk)
